# An adaptive microscope for the imaging of biological surfaces

**DOI:** 10.1038/s41377-021-00649-9

**Published:** 2021-10-07

**Authors:** Faris Abouakil, Huicheng Meng, Marie-Anne Burcklen, Hervé Rigneault, Frédéric Galland, Loïc LeGoff

**Affiliations:** Aix Marseille Univ, CNRS, Centrale Marseille, Institut Fresnel, Turing Center for Living Systems, Marseille, France

**Keywords:** Biophotonics, Confocal microscopy, Wide-field fluorescence microscopy

## Abstract

Scanning fluorescence microscopes are now able to image large biological samples at high spatial and temporal resolution. This comes at the expense of an increased light dose which is detrimental to fluorophore stability and cell physiology. To highly reduce the light dose, we designed an adaptive scanning fluorescence microscope with a scanning scheme optimized for the unsupervised imaging of cell sheets, which underly the shape of many embryos and organs. The surface of the tissue is first delineated from the acquisition of a very small subset (~0.1%) of sample space, using a robust estimation strategy. Two alternative scanning strategies are then proposed to image the tissue with an improved photon budget, without loss in resolution. The first strategy consists in scanning only a thin shell around the estimated surface of interest, allowing high reduction of light dose when the tissue is curved. The second strategy applies when structures of interest lie at the cell periphery (e.g. adherens junctions). An iterative approach is then used to propagate scanning along cell contours. We demonstrate the benefit of our approach imaging live epithelia from Drosophila melanogaster. On the examples shown, both approaches yield more than a 20-fold reduction in light dose -and up to more than 80-fold- compared to a full scan of the volume. These smart-scanning strategies can be easily implemented on most scanning fluorescent imaging modality. The dramatic reduction in light exposure of the sample should allow prolonged imaging of the live processes under investigation.

## Abstract (web summary)

A smart-scanning fluorescence microscope adapts its scanning scheme to the curved surface of embryonic cell sheets. This results in a considerable reduction in light dose without deterioration of the images.

## Graphical abstract

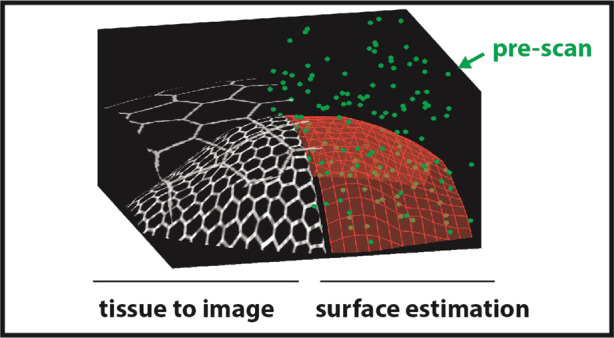

## Introduction

Modern techniques in fluorescence microscopy allow to image entire biological tissues and embryos at diffraction limited resolution^1^ or even sub-diffraction limited resolution^2^. In the widely used laser scanning confocal microscope, a focused laser is scanned throughout the sample to generate a 3D image^3^. The geometry of light excitation is such that planes out of focus are irradiated as much as the imaged focal plane^4^. The integrated light dose impinging on the biological sample then scales with the number of acquired planes required for volumetric imaging. This is a major experimental limitation because light exposure not only causes photobleaching of the sample but also alters cell physiology^5^. In this study, we propose a method to strongly reduce sample irradiation by reducing the number of scanned points of excitation, without loss of resolution.

One means to reduce irradiation of the sample is to adjust light exposure dynamically, changing either the dwell time or the power of excitation at each imaging point. Such a strategy has been implemented with success on different scanning imaging modalities, such as confocal^6^ and two photon fluorescence^7^, stimulated depletion microscopy^8–10^, as well as wide field and structured illumination microscopy^11,12^. In these implementations, the light dose is modulated at each pixel to reach a prescribed signal to noise ratio. These approaches use information at the scale of a single pixel in order to modulate light dose. Thus, scanning of all pixels is required.

**Fig. 1:**
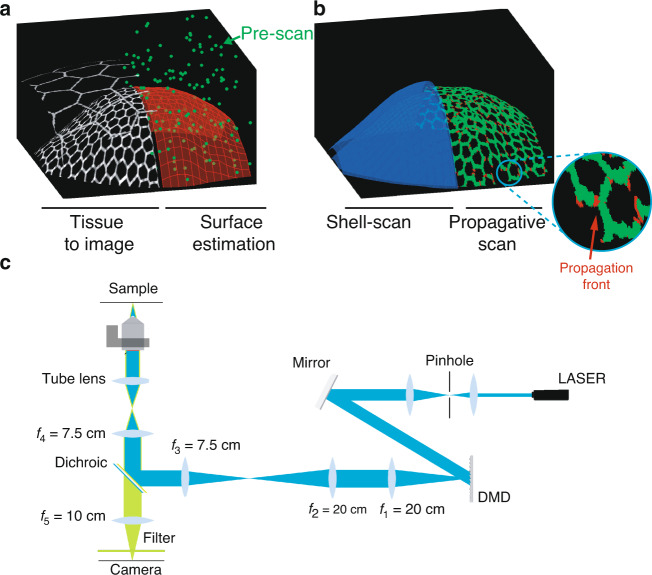
Approach and experimental set-up. **a**, **b** Schematic of the approach. **a** Left: Drawing of a curved epithelial tissue. The tissue may be overlaid by a second epithelium to be discarded by the imaging process. Right: We first estimate the surface of interest (red mesh) using a small fractional pre-scan (green dots). **b** After surface estimation, we either scan a thin shell (blue) around the surface of interest (left side) or we propagate the scanning process along the cell outlines (right side). The zoom in the inset shows the propagation front in red while the previously sample voxels are in green. **c** Experimental set-up

In this study, we drastically reduce the number of scanned voxels in the imaging process by exploiting the higher-order geometrical organization of biological tissues in 3D. A typical example are epithelia, in which cells are organized in cell sheets, and which take a central role in structuring embryos and developing precursors of adult tissues. The physical integrity of an epithelium is ensured by adherens junctions which involve adhesion complexes and the cytoskeleton, organized in a belt-like fashion around each cell. Adherens junctions concentrate a lot of the forces underlying morphogenesis^13^. When imaging epithelial tissues, adherens junctions appear as a mesh of cell-contours lying on the surface of the epithelium (Fig. 1a, left). The fluorescent structures then occupy only a small fraction of the total volume of acquisition. This is all the more pronounced that epithelia are usually curved in 3D, which forces to perform large volumetric imaging, leading to bleaching and phototoxicity. Image post-processing algorithms are also required to extract from the full 3D volume the 2D image lying on the surface of interest for biological analysis^14–18^. A conventional raster scan of the excitation beam is suboptimal to image such sparse structures.

We present a paradigm for scanning fluorescence microscopes, which is adapted to the imaging of curved structures. Our experimental implementation uses a digital micromirror device (DMD) for multipoint illumination, but the principle could be applied to most scanning microscopes, including commercial confocal fluorescence microscopes. The principle is to dynamically adapt the scanning scheme to image epithelial surfaces via an algorithmic search for informative voxels, which correspond in our case to the voxels labelled by the junctional marker (i.e. bright voxels) and distributed along the epithelial surface. Our approach consists in selectively illuminating a sub-volume that closely match the structure of interest. Most importantly, the subvolume must be automatically estimated from a reduced set of measured points that contributes marginally to the integrated light dose.

To define the illumination sub-volume, we present two alternative approaches that interrogate the geometry of the tissue at different scales. The first approach assesses the global scale, estimating high-order organization of the curved surface of the epithelium from a very small set of measured points (Fig. 1a, right). This allows to fully scan only a thin shell around this surface (Fig. 1b, left) to retrieve a 3D image of the tissue at full resolution with a much reduced integrated light dose. Typically less than 5% of the voxels are sampled. The second approach assesses the mesoscopic scale inside the estimated thin shell, limiting the acquisitions to the contour of cells. This is done through an adaptive acquisition which scans the image at informative voxels i.e. those lying on the fluorescent contours of the cells of the epithelial surface, while limiting acquisitions outside of these contours (Fig. 1b, right). The scan-path is generated iteratively in an unsupervised manner allowing to image the tissue of interest with only a fraction of the voxels being actually sampled (~1–2%).

## Results

We built a scanning fluorescence microscope that can acquire an arbitrary set of voxels (see methods and Fig. S1). Briefly, the optical layout (Fig. 1c) uses a targeted illumination operated by a DMD to control point illuminations (Fig. S1a–c). We scan along xy, by turning on micromirrors of the DMD sequentially and along z by moving the objective.

We use the microscope in different scanning modalities. The *full scan* is a conventional scan of the entire sample space. The *pre-scan* is a highly fractional (~0.1%) sampling of the sample space used to explore the geometry of the biological structure of interest. The *shell-scan*, described in the first result-section, limits scanning of the excitation foci to a shell around the biological structure of interest, which was determined from an analysis of the pre-scan. The *propagative scan*, described in the second result-section, restricts even more scanning of the excitation foci to the fluorescent structures along the cell borders using a propagative algorithm within the shell of interest. The shell-scan and the propagative scan have in common that they both start with the estimation of the surface and shell of interest.

The shell-scan and the propagative scan are adaptive smart-scanning strategies that image the biological structure of interest on the surface of the cell sheet while reducing the volume of the scanned path. The consequence of this reduced scanned path is an equally reduced light dose.

### Global scale strategy: Scanning a surface of interest (the shell-scan)

We estimate the biological surface of interest by performing a pre-scan of the sample, collecting only ~0.1% of the voxels. Such a fractional pre-scan is a negligible contribution to the total light dose received by the biological sample. The full method for surface estimation is detailed in the methods section, associated Supplementary Fig. S2, and supplementary text. Briefly, the fluorescence data collected in the prescan is first normalized to remove spatial inhomogeneities of the intensity distribution (equation (1), Fig. S2a, b). We detect bright points from this normalized intensity distribution and remove bright points that are not part of the most densely populated surface through filtering approaches, including local RANSAC polynomial fits on overlapping windows (Fig. S2c–e). A surface modeled as *z* = *Z*(*x*, *y*) is then determined from these points and converted into a thin shell by setting the shell thickness to 3*μ*m, which is slightly larger than the thickness of adherens junctions^19^.

**Fig. 2:**
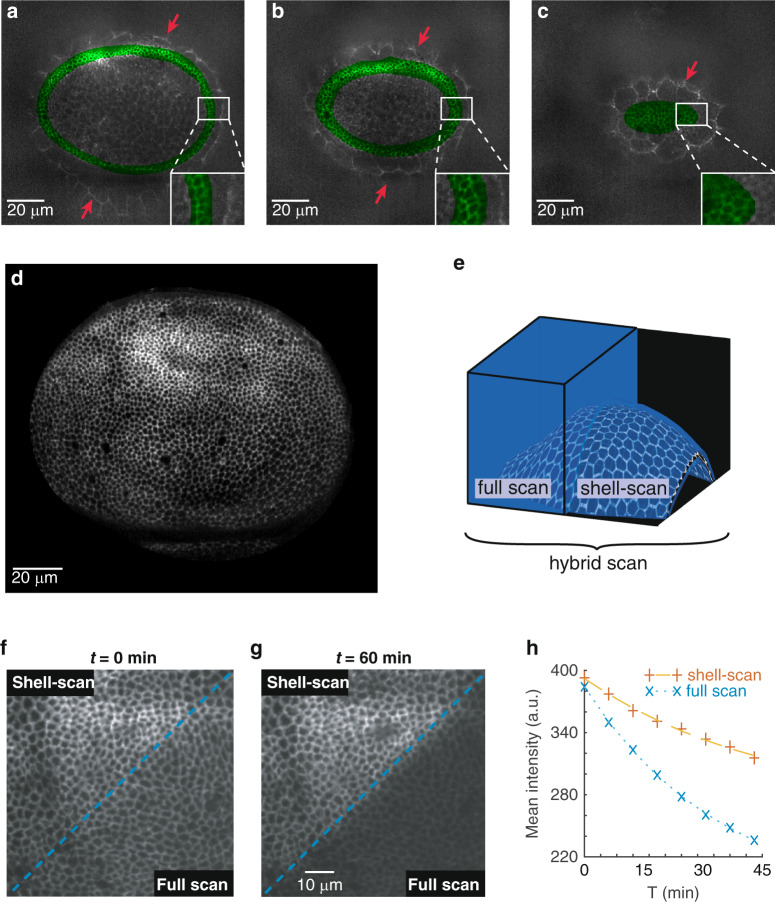
Shell-scan around the surface of interest. **a–c** Overlay of full scan XY sections of the tissue (gray) with the estimated shell (green) at 3 different planes. Imaginal cells are well encapsulated by the shell, while cells of the overlying peripodial membrane (red arrows) are properly discarded. **d** Maximum intensity projection of the normalized signal (equation (1)) acquired with the shell scan on a wing imaginal disc. **e**–**h** Comparing image acquisition with shell scan and full scan. A hybrid scanning strategy is performed to simultaneously image half of the tissue in full scan and half in shell-scan (**e**). **f**, **g** Maximum intensity projection. While the two adjacent zones are initially indistinguishable (**f**), bleaching is more prominent in the full scan zone after 60 min of continuous imaging (**g**). **h** Quantification of the bleaching process showing the temporal evolution of the maximum intensity projection averaged over the full scan or shell-scan regions

To assess the quality of the estimation, we perform a full scan of the entire volume to provide a ground truth, which we superimpose with the mask of the shell for comparison. Figure 2a–c clearly shows that the mask of the shell encapsulates tightly the cell contours of the actual wing disc epithelium which lie in the focal plane. Other sources of signal are rejected from the shell. This includes the peripodial membrane, an epithelium facing the wing imaginal disc in a luminal configuration, which is less populated in bright points (red arrows in Fig. 2a–c). Further quantitative assessments of the fit are given in the last result section (Fig. 4), and in the supplementary section (Fig. S4).

A high resolution image of the curved epithelial surface with an improved photon budget is then obtained by scanning the excitation foci exclusively in the shell around the surface—the shell-scan. Figure 2d shows the maximum intensity projection of the normalized signal (equation (1) in the methods) obtained with this “on the fly” shell-scan of a wing imaginal disc. In this example, only ~3% of the voxels were sampled, leading to more than a 30-fold reduction in light dose compared to a full scan. Table 1 recapitulates scanned-volume reduction of the shell-scan. A more systematic exploration of scanned volume reduction is also performed in the last result section.

**Table 1:** Reduction in scanned volumes in the shell-scan and propagative scan for the tissues analyzed in Fig. 3.

	Full scan	Tight bounding box	Shell-scan	Propagative scan
small cells: wing disc of Fig. 2d	100%	27%	3%	N/A
small cells: wing disc of Fig. 3d	100%	32%	3%	1.5%
large cells: epidermis of Fig. 3e	100%	94%	34%	1.2%

The contribution in the reduction from scanning a tight bounding box around the tissue is also added. Other comparisons are provided in Figs. S5, S6, and S7

To highlight the benefit of the shell-scan, we performed a hybrid scan of a wing imaginal disc: half of the sample was imaged via the shell-scan and the other half via the full scan (explanatory drawing in Fig. 2e). At the first acquisition, the two regions are indistinguishable on the resulting image (Fig. 2f), even though the shell-scan region used a fraction of the light dose of the full scan. Notably, the thickness of the cell-cell interfaces, estimated by fitting the cross profile of 50 interfaces on each side with a gaussian profile, were not significantly different (shell-scan: 3.2pixel ± 0.5(std), full scan: 3.2pixel ± 0.4(std)). This is expected from the fact that the same imaging process is performed on either side, governed by the same point spread function. Most importantly, after 60 min of constant imaging, the shell-scan region experienced much less photobleaching (Fig. 2f–g, quantifications in Fig. 2h). This proves the interest of the proposed shell-scan approach in terms of bleaching reduction, a major issue when imaging embryos and developing tissues, and illustrates the versatility of the built microscope on which any acquisition path can be implemented. To illustrate how the difference in light dose and fluorescence dynamics can impact the characterization of biological processes, we quantified local cell movements using particle image velocimetry (PIV, Fig. S3a). PIV uses cross correlation in small overlapping regions to compute a field of displacement from the analysis of two successive images^20^. We measure with a better accuracy the displacements in the shell-scan region (Fig. S3b, c). Computed displacements also have a higher average value (Fig. S3d), which may result from an underestimation of the displacements in the bleached region or an effect of the light dose on cell physiology.

To conclude, we have drastically reduced volume acquisition by automatically adapting the scan path to the sample under investigation. The sample then benefits from a much reduced light dose without loss in imaging resolution.

### Meso-scale strategy: scanning the outline of cells with a propagative algorithm (the propagative scan)

Even with the shell-scan, the imaged fluorescent structures occupy only a small fraction of the scanned volume. In the case of adherens junctions, the structure of interest is located at the cell periphery. Scanning the center of cells does not contribute significant fluorescence from the structure of interest, but it does potentially affect fluorophores and cell physiology outside of the imaged plane. Here, in the second strategy, we use a propagative algorithm that reduces acquisitions in these non-fluorescent voxels and scans iteratively a subvolume that encapsulates as closely as possible the cell outlines.

The propagative scan, like the shell-scan, starts with the estimation of the surface of interest, and corresponding shell by setting again a thickness of 3 μm, with the aforementioned algorithm. This time, not all the voxels of the shell will be scanned by the excitation foci. First, a new small random set of voxels is acquired within the shell to provide additional seeds of propagation. Then, an extrapolation based on the nearest acquired neighbor is performed in non-scanned territories. Only the voxels that are predicted to be bright are then scanned (detailed in the methods section). This propagative scan automatically stops when the propagation has converged, which corresponds to the first iteration without new acquisitions. It can also be manually stopped earlier, as the number of new acquisitions decreases rapidly.

**Fig. 3:**
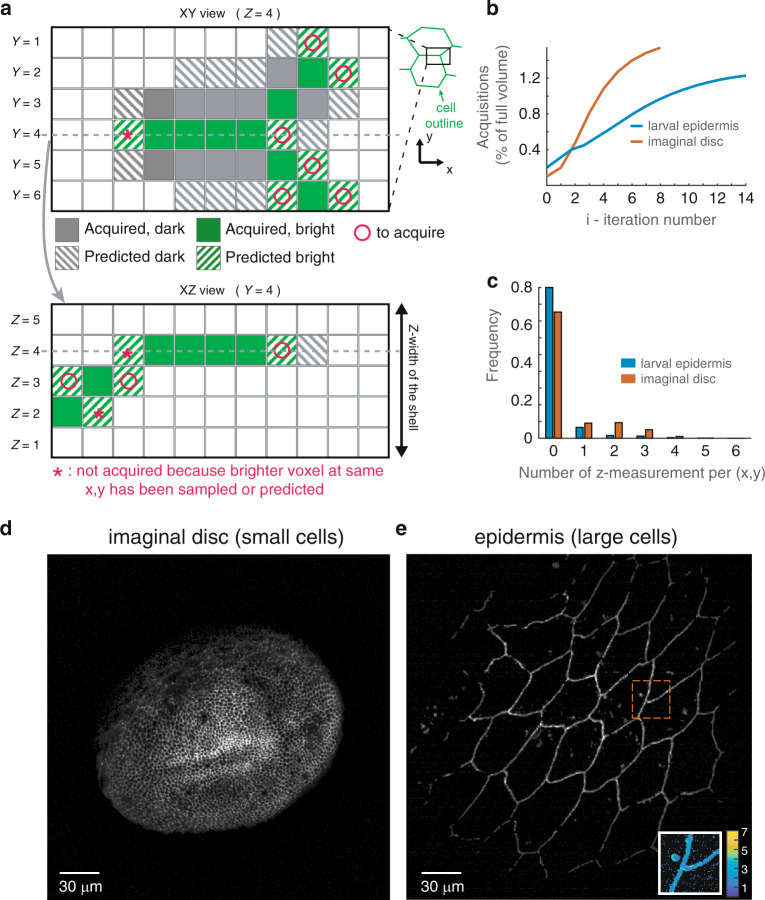
Propagative scan within the estimated shell. **a** voxel-level schematic of the algorithm. Using the information from previously acquired voxels (which may be bright or dark voxels), a prediction (hatched coloring) based on the nearest neighbors is made for neighboring pixels in each layers of the shell, and at a distance *β* = 1 in this example. Two corresponding XY and XZ sections are shown. The signal will be acquired only in the voxels that are predicted to be bright (o). Moreover, on the XZ section, voxels that are predicted to be bright, but for which a brighter voxel has already been acquired or predicted are not sampled (*). **b** Number of acquisitions as a function of iteration number. **c** Number of acquisitions performed along *z* (including the initial pre-scan) for each *x*, *y* using the propagative imaging (*β* = 1 pixel): no acquisitions have been performed for more than 75% of the *x*, *y* coordinates in the case of the small imaginal disc cells and 90% for the large epidermis cells. **d**, **e** Maximum intensity projection of the shell at the end of the propagative imaging for the imaginal disc (**d**) and the epidermis (**e**). An inset in (**e**) maps the number of acquisitions performed along *z* for a small region of interest (outlined in red in the main image). Junctions and endocytic vesicles are fully sampled, while the rest is only sparsely sampled

Such a simple algorithm based on nearest neighbor is well suited for cell contour detection as it naturally tends to follows lines. We applied the strategy on two different tissues: the wing imaginal disc, constituted of very small cells (~2–3 μm —some cells are less than 6 voxels in diameter), and the larval epidermis, constituted of large cells (~25 *μm* in diameter). The propagative algorithm reaches ~90% of completion after only 6 iteractions in the wing disc and 12 iteractions in the epidermis (Fig. 3b). A good fraction of (x, y) positions are not sampled at any z-position (90% in the epidermis, Fig. 3c), as the algorithm efficiently avoids scanning uninformative voxels. The resulting projections (Fig. 3d–e) are very similar to what can be obtained with the shell-scan or full scan. Notably, in the case of the epidermis, not only were the outline of cells imaged, but an endocytic punctate cadherin pool was also targeted by the algorithm (see the inset in Figure 3e, which maps the number of z-samples inside a small region of interest). The approach can thus be used to image complex cellular processes such as the recycling of adherens junction components.

To further quantify the benefit of the propagative scan, we measured the size of the scanned volumes obtained with different scanning strategies, which are presented in Table 1. On the wing imaginal disc, the propagative scan represents only a moderate 2-fold improvement in light dose with respect to the shell-scan, reaching 1.5% of full scan. The moderate improvement is explained by the fact that the length scale over which the algorithm operates compares with the cell diameters. On the larval epidermis, the propagative scan was a 28-fold reduction from the shell-scan, reaching ~1.2% of full scan (while the shell was ~34% of full scan in this fairly flat tissue). This shows that on sufficiently large cells, the propagative scan provides a very large reduction in light dose compared to the shell-scan, and even more so compared to a full scan acquisition.

### Quantifying the accuracy of the shell-scan and propagative scan

**Fig. 4:**
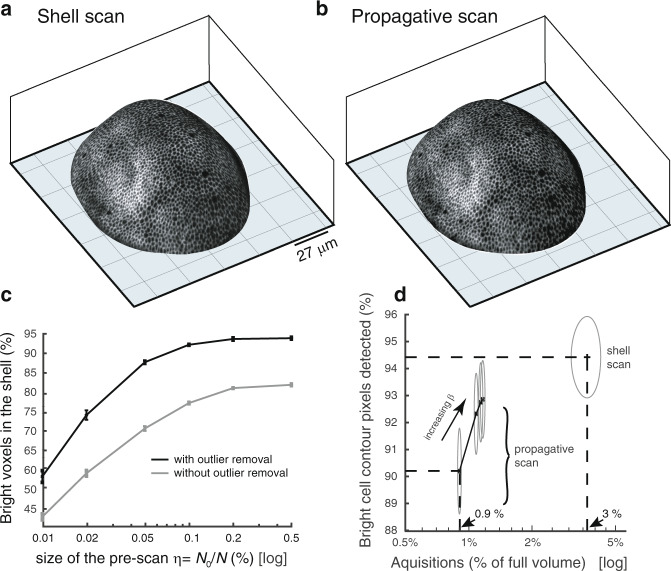
Accuracy of shell estimation and ability of bright contour acquisition. **a**, **b** 3D visualizations of emulated shell-scan and propagative-scan (*β* = 5) using raw data from a full scan acquisition. **c** Goodness of the shell estimated as a function of the size of the pre-scan. The goodness is measured from the percentage of epithelial bright voxels (i.e. bright voxels that are in the ground truth shell) that fall in the estimated shell (the objective being to maximize this quantity). The result is shown with and without outlier removal in the surface estimation step. **d** Goodness of cell contour delineation with the shell-scan and propagative-scan, as a function of the number of acquisitions required. For the propagative-scan, results with different NN maximal prediction distances *β* are shown (*β* = 1; 3; 5; 10). All points plotted on these curves correspond to the average of the result over 50 different random pre-scans (with error bars in (**c**) and the height and width of ellipses in (**d**) set to their standard-deviation)

How do the shell and propagative scans compare with a full scan on a quantitative basis? To address this, we performed a full scan image of a wing imaginal disc. From this, we determined a ground truth epithelial shell with a manually curated surface-fit, using a shell-thickness of 3*μ*m as in previous acquisitions. We then emulated the shell-scan and propagative-scan from the raw data for comparison with the ground truth image on a voxel by voxel basis. Figures 4a–b reports the 3D visualizations of emulated shell-scan and propagative-scan by projecting the 2D maximum intensity projections on the 3D surfaces. The two images are very similar on qualitative ground. It then seems that most of the bright cell contours visible in the shell-scan have been captured in the propagative scan. Figure 4c reports the quantification of the accuracy of surface determination by counting the percentage of bright voxels in the ground truth epithelial shell that are present in the estimated shell $\widehat{{{{\mathcal{S}}}}}$ as a function of the pre-scan sampling ratio (*η*). As expected, increasing the size of the pre-scan increases the percentage of epithelial bright voxels in $\widehat{{{{\mathcal{S}}}}}$. It also appears that *η* = 0.1% -the setting used throughout this study for wing discs- is a good trade-off between quality of the results and number of acquisitions, as the curve tends to plateau beyond that point. Moreover, when we omit the step of outlier removal (described in the methods section and in the supplementary section) in the surface estimation step, the estimated surface is deteriorated. Thus, the percentage of epithelial bright voxels in $\widehat{{{{\mathcal{S}}}}}$ is significantly reduced (gray curve in Fig. 4c). This confirms the need for a technique which is robust to the presence of outliers in the surface estimation step. Other parameters of surface determination have also been systematically investigated in Supplementary Fig. S4a–b. This includes the number of overlapping windows in which the polynomial fits are performed, and the severity of the outlier removal step. In short, in presence of smooth surfaces the number of overlapping windows does not significantly influence surface estimation when we use a sufficiently strict outlier removal step. Moreover, although local (*x*, *y*) polynomial functions are used, results in Supplementary Fig. S4c–d confirm that this approach can still be employed in presence of a non-polynomial step, as soon as sufficiently small sliding windows are used (at most twice larger than the step-width), even when more than 2/3 of bright points correspond to outliers. Finally, Fig. 4d shows a comparison of the goodness of cell outlining in the two scanning strategies, as a function of the number of acquisitions. More specifically, after performing a maximum intensity projection in the ground truth shell, we measured how many of its bright points were also detected as bright in the maximum intensity projections of the shell-scan and the propagative-scan (bright cell contour detected). In the case of the propagative-scan, results for different values of nearest neighbor prediction maximal distance *β* are shown (*β* ranging from 1 to 10 pixels). Figure 4d confirms the complementarity of the two strategies. Both of them allow to see cell shapes while scanning only a few percent of the sample space. The shell-scan captures a more complete set of bright points compared to the propagative scan but at the expense of a larger fraction of acquired voxels.

## Discussion

We have built a scanning fluorescence microscope dedicated to the imaging of epithelial tissues. Our set-up adapts its scanning scheme to the morphology of the sample. Our novel approach allows to reduce the scan volume of large samples by nearly ~99% in some instances. The reduction could be larger for highly curved samples comprising large cells when imaging cell contours. The microscope uses a targeted illumination operated by a DMD to control point illuminations, while virtual pinholing ensures optical sectioning. We propose two complementary scanning strategies, which rely on the progressive delineation of structures of interest based on fractional acquisitions of the volume. Notably, both methods start with an initial prescan of about one thousandth of the sample space in order to estimate the surface of interest.

The first strategy processes information at the global scale, i.e. of the overall shape of the tissue, to reduce the scanned volume and thus the light dose. The acquisition is limited to a thin shell encapsulating the surface of interest. The second strategy processes information at the meso-scale, focusing on the contour of cells. The scanning scheme of the latter is far away from a conventional raster scan in that it progressively delineates and acquires cell outlines. How much exactly each method reduces the light dose depends on the shape of the tissue and on the size of cells within the tissue. Both approaches are particularly well suited for tissues with a low surface to volume of bounding-box ratio -typically cups, spheres or ellipsoids.

When should either of the proposed methods be used? On qualitative grounds, the shell-scan provides greater details of cell structures. The propagative scan, in essence, ignores cell parts considered non-informative. It thus provides a less detailed representation of cell structure (Fig. 4). However, the information loss can be minimal and the benefit in light dose very significant. The extent of this benefit will be set by the characteristic size of the imaged cells compared to the resolution of the microscope. The small size of imaginal-disc cells made this added benefit moderate, while the large size of epidermal cells allowed a 30 fold reduction in light dose compared to the shell-scan (see Table 1). Of note, our current set-up under-samples the diffraction limit (voxel size ~0.27 μm in x,y). One can expect an improvement of the light dose in the propagative scan on the small wing disc cells with a better resolved microscope. Finally, one important difference between the two approaches is that the propagative scan relies on multiple camera-exposures to allow for the iterative process, resulting in a slower acquisition. This issue could be alleviated in a single point scanning scheme, where imaging rate is essentially set by the total length of the scanning path. The propagative scan may then become faster, provided real-time data analysis can take place in parallel of acquisition.

Beyond our DMD-based, multipoint illumination set-up, the proposed strategies could be applied to any fluorescence imaging modality where it is possible to spatially pattern illumination. This includes the confocal laser scanning microscope (CLSM), a widely used technique for tissue imaging. A targeted illumination can be obtained on a CLSM through the combined real-time measurement of the laser position (a usual read-out from galvanometric scanners) and the digital modulation of laser power. The algorithms used in our study should also be directly applicable to CLSM: in Supplementary Figs. S5, S6, S7, we successfully emulate the shell-scan and propagative scan on images of imaginal discs acquired with a spinning disc confocal microscope using the same parameter settings as Figs. 2, 3, 4. Smart scanning schemes could also be helpful in the context of non-linear contrasts. There, the stress imposed on the fluorophores is mainly constrained to the plane of imaging, which may reduce the need for our strategy to control photobleaching. However, the reduced light dose of the shell-scan and the propagative scan may help to restrain thermal effects associated with near infrared pulsed light absorption, observed both in the non-linear^21,22^ and linear regime^23^. The reduced scan path of our strategies would also help speeding-up non-linear microscopy, which tends to be slower than linear microscopy.

In this paper, the scanning schemes are based on a preliminary estimation of the surface of interest. One strength of the proposed approach is its robustness to the presence of other less populated surfaces and scattered bright points, without requiring the acquisition of more than ~0.1% of the voxels to determine this surface. Furthermore, most of the unknown variables of the approach are automatically estimated using only the few voxels acquired in the pre-scan, thus avoiding the need for a pre-calibration step of the algorithm. One key parameter is nevertheless the smoothness of the imaged surface of interest, which is set by the typical size at which the surface can be approximately fitted with a second order polynomial fit. This parameter is known in most situations and the algorithm is relatively robust to its setting (see Fig. S4b). Some tissues can have large enough spatial irregularities for manual tuning of window size to be required (Fig. S6). Nevertheless, several observations indicate that our algorithm for surface computation could be used in many biological contexts without modifications. First, many embryos or developing tissues are smooth surfaces showing little irregularities. Being a simple surface, is one of the reasons to become an experimental model system in morphogenesis, if only to allow for good optical imaging. Second, we demonstrated that the algorithm can withstand surface irregularities such as folds or tears (Fig. S6). Third, it can also withstand more complex topologies, as illustrated in Fig. S7, where the shell-scan and propagative scan were successfully emulated in a tissue with two surfaces. One stimulating perspective will however be to extend the proposed approach to deal with other kinds of surfaces, such as closed surfaces for which a modeling as a function of x,y is not adapted anymore.

In the propagative acquisition strategy, a simple prediction in the nearest neighbor sense has been used. Although promising results have been obtained, a possible improvement would be to use more sophisticated approaches to iterate the aquisition. For example, in refs. ^24,25^, data based learning approaches are used to decide where new acquisitions should be performed to improve reconstruction.

Our work has focused on the acquisition of a single image of a 3D epithelial structure. Time sequences (Fig. 2f–g) just repeat the process. Currently, the numerical processing (in Matlab) requires only ~4 s to estimate the surface from the prescan, and ~0.9 s for each step of the propagative scan in case of the example of Fig. 4, and respectively ~12 s and ~1.3 s on the emulated image of Fig. S5 (computation times obtained on a MacBook-pro labtop, with 2.8 GHz Intel Core i7 with 16 Go RAM). In comparison with the typical time required to scan an equivalent volume with a confocal (~50 s for 50 × 1000 × 1000 voxels with μs dwell time) and the similar requirements on our set-up, the overtime associated with the computation is not significant. Speeding up computation, which has not been fully optimized, may be as simple as using a computing workstation instead of the current laptop, or compiling our Matlab script. A natural extension of our work will be to address how temporal redundancy could be used to optimize the scanning strategy in a time sequence. Using the information extracted from a given time point as a prior for the next time point could considerably increase the speed of the procedure.

A method of choice when it comes to light dose reduction is light sheet fluorescence microscopy (LSFM)^1,26,27^. LSFMs offer unsurpassed speed and depth of imaging on large samples, while being very efficient in term of light dose^28^. Nevertheless LSFM and CLSM correspond to different applications. A user will typically use CLSMs for high resolution imaging on fairly shallow samples and LSFM for lower resolution imaging on larger samples. The reduced light dose of the shell and propagative scans may allow to extend the application range of CLSM in live imaging when LSFM cannot be used.

Lastly, several recent improvements in fluorescent imaging have emerged that could be combined with our approach. First, denoising techniques allow to image tissues at low excitation power using post acquisition denoising to compensate for the low SNR^5,29^. This could be used to further reduce irradiation levels in our microscope. Second, faster imaging and compression of the signal from our 2D manifold in a 3D space could be achieved through extended depth of field using electrically tunable lenses as in ref. ^30^. Finally, photon reassignment^31,32^ or learning techniques^33^ could be combined with our approach to improve lateral resolution.

To conclude, we developed a robust smart-scanning technique that can easily be implemented on most existing fluorescent microscopes. Our technique dramatically reduces sample exposure to illumination, thus allowing prolonged imaging of the live process under investigation. Our work is in line with the endeavor to build “smart and gentle microscopes”^26^ for live imaging of sensitive biological samples.

## Methods

### Experimental set-up

We built a scanning microscope that can acquire an arbitrary set of voxels. The set of voxels and the scanning schemes are driven by information processing algorithms. The set-up (Fig. 1c) aims at illuminating selected voxels with a Digital Micromirror-Device (DMD, Vialux, V9601) in a similar fashion to^12,32,33^. A conventional epifluorescence microscope (Zeiss, Axiovert 200M) images the sample plane onto an sCMOS camera (Orcaflash, Hamamatsu) through an objective (Zeiss, C-apochromat, 40×, 1.2NA), tube lense and additional 4f system. A 488 nm laser (Cobolt 06 serie MLD, 200mW), collimated and expanded, is shined to the DMD chip surface. The DMD acting as a grating, diffraction effects must be considered; the laser beam incident angle is then set to select the brightest order. The DMD is placed in a plane conjugated to the sample plane such that any ON/OFF matrix loaded in it creates an illumination pattern in the sample plane, convoluted by the point spread function (PSF) of excitation (Fig. S1). Overall, the magnification of the excitation and imaging branches are such that one micromirror (10.8 *μ*m) projects into a square of 270 nm in the sample plane, and one pixel of the camera projects into a square of 117 nm. We spatially mapped the DMD lattice onto the camera sensor by registering numerous known patterns illuminating a homogeneously fluorescent sample. With such a map, if a pixel of the DMD is switched on, the position on the camera of the expected signal response is known. To scan along the xy-plane, the excitation foci are moved by sequentially turning on neighboring micromirrors of the DMD (Fig. S1a–c). To increase the speed of imaging, we use multiple foci arranged in a hexagonal lattice. Scanning along z is performed by translating the objective with a piezo electric element. Optical sectioning is performed numerically by simply keeping the signal near the conjugated point of the foci on the camera and discarding the out of focus blur further away from the conjugated points^32,34^ (Fig. S1d). To avoid crosstalk between illumination points, we decompose the set of pixels to acquire into a batch of acquisition masks where illumination points are separated by a minimum distance of 5.5 μm. 3D imaging is performed by serial imaging of multiple planes. Voxel size is 0.27 μm × 0.27 μm × 0.5 μm (x, y, z). The typical full stack 3D voxel space is 915 × 915 × 50.

### Algorithms

#### Identification of a surface of interest from a fractional prescan

We adapt scanning strategies to curved surfaces. For this, we determine the surface of interest from the sampling of only a small fraction *η* of the voxels.We typically use *η* = 0.1% for most tissues. This fractional pre-scan is generated projecting random lattices of point illumination in a subset Ω_0_ of the full voxel space Ω, such that *η* = card[Ω_0_]/card[Ω], where card stands for the cardinal, the number of elements of an ensemble.

We first correct spatial inhomogeneities of the background signal, i.e. the signal coming from voxels that are not enriched in green fluorescent protein (GFP). This is done by introducing a normalized signal,

1 $${\widehat{r}}_{[{{{\Omega }}}_{0}]}(x,y,z)=\frac{s(x,y,z)-{\widehat{a}}_{[{{{\Omega }}}_{0}]}(x,y,z)}{{\widehat{a}}_{[{{{\Omega }}}_{0}]}(x,y,z)\,{\widehat{\sigma }}_{[{{{\Omega }}}_{0}]}}$$

where *s*(*x*, *y*, *z*) is the measured signal, ${\widehat{a}}_{[{{{\Omega }}}_{0}]}(x,y,z)$ is an estimation of the background spatial inhomogeneities and ${\widehat{\sigma }}_{[{{{\Omega }}}_{0}]}$ is an estimation of the standard deviation of $s(x,y,z)/{\widehat{a}}_{[{{{\Omega }}}_{0}]}(x,y,z)$ on the background. Both ${\widehat{a}}_{[{{{\Omega }}}_{0}]}(x,y,z)$ and ${\widehat{\sigma }}_{[{{{\Omega }}}_{0}]}$ are determined using only the fractional pre-scan Ω_0_ (see the supplementary section for details). Through this normalization, the histogram of ${\widehat{r}}_{[{{{\Omega }}}_{0}]}$ is expected to approximately fall down on an heavy-tailed Gaussian-like distribution (Fig. S2a and b). The Gaussian shape of the distribution, of zero-mean and unit variance, should correspond to background voxels, while the heavy tail is related to bright points (arrow head in Fig. S2b and its inset). We can then detect bright points using a pure significance test^35^, as ${\widehat{r}}_{[{{{\Omega }}}_{0}]}(x,y,z) \,>\, T$. The threshold *T* is set by the chosen probability of false alarm (pfa), i.e. the probability to detect a background voxels as a bright point. Assuming ${\widehat{r}}_{[{{{\Omega }}}_{0}]}(x,y,z)$ has a normal distribution on background voxels, $\,{{\mbox{pfa}}}\,=\frac{1}{2}\left[1-\,{{\mbox{erf}}}\,(T/\sqrt{2})\right]$, where erf is the error function. For this whole study we chose a pfa of 1%, which corresponds to *T* ≈ 2.33. In the example of Fig. S2d, among the 0.1% ≈ 40000 points acquired during the pre-scan, ~900 bright points distributed in the volume are then detected as bright. When imaging very large cells such as in the epidermis, very few voxels are bright. We then used *η* = 0.2% to increase the number of bright points — a value which could also have been used successfully in the wing disc, since performance naturally increases with *η* (see Fig. 4c). Note that, instead of fixing *η* from a priori knowledge on the size of the cells, an interesting perspective would be to progressively increase *η* from very low value, until a sufficient number of bright points have been detected to be able to accurately estimate a surface.

In a second step, we interpolate the epithelial surface modeled as *z* = *Z*(*x*, *y*), using the detected bright points. Since the epithelium is not infinitely thin, the bright points are not located exactly on this surface, but at a small distance from it. To cope with this source of noise on location, but also with outliers due to false alarms or to fluorophores located outside the surface of interest (e.g. on another less populated surface as shown in Fig. 1a), we use local second order polynomial fits of the bright point z-coordinates, combined with RANSAC-based outlier removal^36^. The fits are estimated in overlapping windows (with width a third of the image width) and are then fused (see the supplementary section and drawing of Fig. S2c). This allows us to keep only the bright points that are close to the most populated surface (inliers) and to denoise their z-coordinates before interpolation, which is all the more important that Ω_0_ contains few points. Figure S2d shows the result of this classification of bright points of Ω_0_ between inliers and outliers. A surface $\widehat{Z}(x,y)$ can then be estimated at every point of the image using a simple bi-cubic harmonic spline interpolation^37^ from these denoised inlier points. The resulting surface is shown in Fig. S2d. Once the surface of interest $\widehat{Z}(x,y)$ is determined, it is converted into a thin shell $\widehat{{{{\mathcal{S}}}}}=\{(x,y,z)\ {{{\rm{so}}}}\ {{{\rm{that}}}}\ |z-\widehat{Z}(x,y)|\le \varepsilon /2\}$ by setting a thickness along *z* of *ϵ* = 3 *μm*, which is slightly larger than the thickness of adherens junctions^19^.

#### Propagative scan of cell outlines

Once the surface of interest has been estimated, a first strategy, the shell-scan (see the results section), scans a thin shell around the estimated surface of interest. In an alternative strategy, we further reduce the scanned volume by acquiring signal along the cell contours within the shell. This second approach (the propagative scan) is iterative. For each iteration, the objective is to determine where to focus acquisitions using the voxel intensities acquired at previous iterations. Let us denote Ω_*i*_ the set of all voxels acquired until the end of iteration i with *i* ≥ 0 (iteration i = 0 corresponds to the pre-scan used for the surface estimation). We perform at iteration i = 1 a new random scan of *N*_0_ voxels located inside the shell because only a few of the *N*_0_ = card[Ω_0_] points acquired during the pre-scan fall inside the shell. This additional scan, although optional, improves the quality of the image obtained at the end of the iterative strategy. Then, to determine which voxels should be scanned at iteration *i* > 1, an extrapolation in the nearest neighbor sense of the normalized intensity ${\widehat{r}}_{[{{{\Omega }}}_{0}]}$ (obtained from equation (1) using parameters ${\widehat{a}}_{[{{{\Omega }}}_{0}]}$ and ${\widehat{\sigma }}_{[{{{\Omega }}}_{0}]}$ estimated with the pre-scan) is applied at a small distance from Ω_*i*−1_(Fig. 3a). More precisely, for any voxel (*x*, *y*, *z*) in the shell unexplored at iteration *i* − 1 (i.e. outside Ω_*i*−1_), the predicted normalized intensity *r*_*i*_(*x*, *y*, *z*) at iteration *i* is set to ${\widehat{r}}_{[{{{\Omega }}}_{0}]}({x}_{N},{y}_{N},{z}_{N})$, with (*x*_*N*_, *y*_*N*_, *z*_*N*_) the coordinates of its nearest neighbor (NN) in Ω_*i*−1_. In order to take into account the curvature of the surface, the shell is decomposed into curved layers of one voxel depth along *z* which are all parallel to the surface. This is a natural way to define the coordinates of points in the reference frame of the shell -using as a reference the *z* coordinate of the extracted surface at the same (*x*, *y*) position. The NN (*x*_*N*_, *y*_*N*_, *z*_*N*_) of (*x*, *y*, *z*) is then defined as the point in Ω_*i*−1_ that is in the same layer as (*x*, *y*, *z*) (and thus in the shell) and at smallest xy-Euclidian distance $d(x,y,{x}_{N},{y}_{N})=\sqrt{{(x-{x}_{N})}^{2}+{(y-{y}_{N})}^{2}}$. Note that this NN point can be non unique: in that case, the point with highest normalized intensity is chosen. To focus acquisitions on bright points, new acquisitions are then performed only on points predicted to be bright points, i.e. unexplored points in the shell for which *r*_*i*_(*x*, *y*, *z*) > *T*. Moreover, to further reduce the number of acquisitions, if several points with same *x*, *y* coordinates (but different *z*) have to be acquired at iteration *i*, only the point with highest prediction *r*_*i*_(*x*, *y*, *z*) will be acquired, provided no other voxel with higher normalized intensity has already been acquired in the shell for same *x*, *y*. NN predictions being often non relevant at high distances, points at a distance *d*(*x*, *y*, *x*_*N*_, *y*_*N*_) > *β* from their NN are not acquired. The influence of this parameter is addressed in Fig. 4d.

### Preparation of biological samples

All observations were performed on living Drosophila tissues, using an E-cadherin:GFP knock-in to image adherens junctions^38^. We performed image acquisitions on two tissues of the developing Drosophila. The first imaged tissue is the wing imaginal disc, a precursor epithelial tissue inside the larva which eventually develops into the adult wing, hinge, and thorax. It is a widely used model system in developmental biology^39^. Ex-vivo cultures of wing imaginal discs were performed as in ref. ^14^. Briefly, living tissues were dissected from late third instar larva using a stereo-microscope, and cultured in a drop of Grace’s insect medium (sigma) in a glass bottom petri-dish. The second imaged tissue is the larval epidermis, a monolayer of epithelial cells which adhere to the cuticle of the larvae and perdures until the early stages of pupal development. We imaged the epidermis in vivo, on late wandering stage larvae. The larvae were anesthetized to prevent muscle contractions, following the protocol of ref. ^16^. These two tissues are interesting limit cases to test our approaches: wing disc cells are very small (~2–3 μm in diameter), while cells of the larval epidermis are large (~25 μm).

## Supplementary information

supplementary material

## Data Availability

A Matlab implementation of the algorithm is available at https://www.fresnel.fr/perso/galland/LSA2021/.
