# Supplementary material for: An adaptive microscope for the imaging of biological surfaces
Source: Light Sci Appl. 2021 Oct 7;10:210. doi: 10.1038/s41377-021-00649-9 (PMC8497591; doi:10.1038/s41377-021-00649-9)
Supplement: Supplementary file 1 — supplementary material [file 41377_2021_649_MOESM1_ESM.pdf]

Supplementary information for: An adaptive microscope for the imaging of biological surfaces

Faris Abouakil[1], Huicheng Meng[1], Marie-Anne Burcklen[1], Hervé Rigneault[1,2], Frédéric Galland[1,*], and Loïc LeGoff[1,*]

[1]Aix Marseille Univ, CNRS, Centrale Marseille, Institut Fresnel, Turing Center for Living Systems, Marseille, France
[2]Lightcore Technologies, 35 rue d'Antibes 06400 Cannes, France
[*]frederic.galland@fresnel.fr, loic.le-goff@univ-amu.fr

ABSTRACT

Scanning fluorescence microscopes are now able to image large biological samples at high spatial and temporal resolution. This comes at the expense of an increased light dose which is detrimental to fluorophore stability and cell physiology. To highly reduce the light dose, we designed an adaptive scanning fluorescence microscope with a scanning scheme optimized for the unsupervised imaging of cell sheets, which underly the shape of many embryos and organs. The surface of the tissue is first delineated from the acquisition of a very small subset ($\sim 0.1\%$) of sample space, using a robust estimation strategy. Two alternative scanning strategies are then proposed to image the tissue with an improved photon budget, without loss in resolution. The first strategy consists in scanning only a thin shell around the estimated surface of interest, allowing high reduction of light dose when the tissue is curved. The second strategy applies when structures of interest lie at the cell periphery (e.g. adherens junctions). An iterative approach is then used to propagate scanning along cell contours. We demonstrate the benefit of our approach imaging live epithelia from Drosophila melanogaster. On the examples shown, both approaches yield more than a 20-fold reduction in light dose -and up to more than 80-fold- compared to a full scan of the volume. These smart-scanning strategies can be easily implemented on most scanning fluorescent imaging modality. The dramatic reduction in light exposure of the sample should allow prolonged imaging of the live processes under investigation.

List of Supplementary material

Supplementary figures
Supplementary text

Supplementary figures

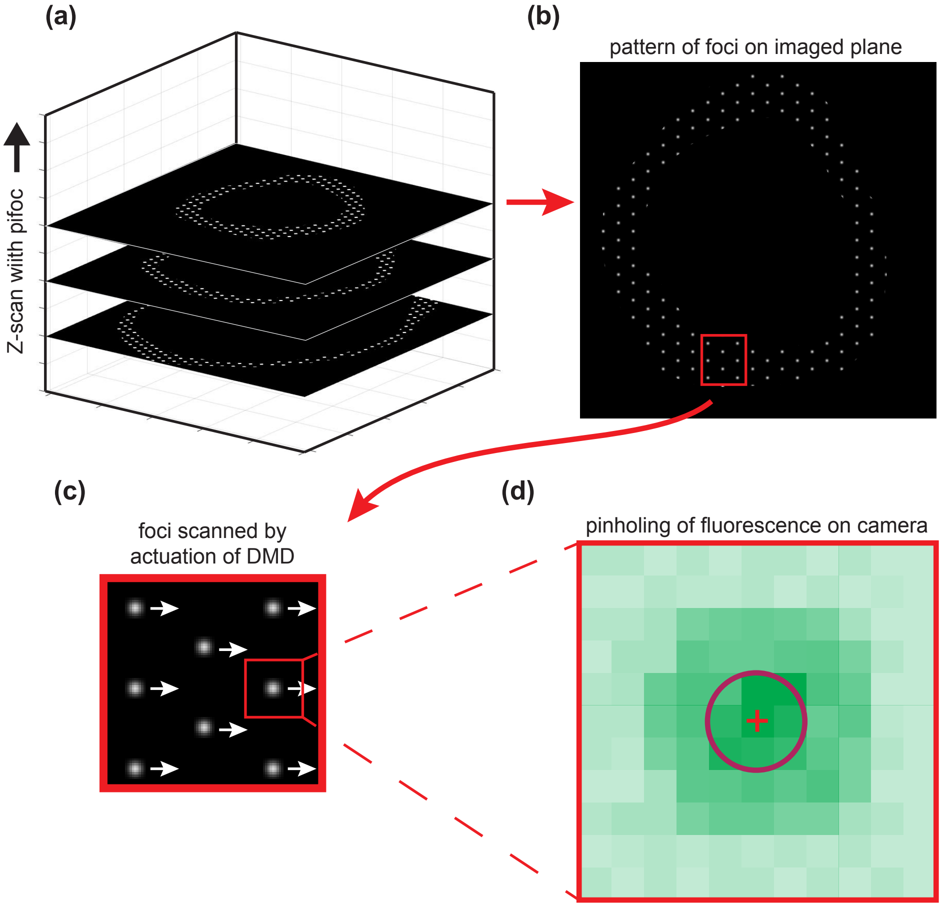

Figure S1. Scanning process. (a) Excitation foci illuminate the sample plane by plane. The shape of the shell can be guessed from the 3 planes shown (out of typically 50). (b) In one plane of excitation, the pattern of foci is a hexagonal lattice masked by the enveloppe of the shell at this plane. (c) To scan along the xy-plane, the excitation foci are moved by sequentially turning on neighboring micromirrors of the DMD. (d) To retrieve sectioning, the small regions (red circle) near the point conjugated to the excitation foci (cross) are kept, while the signal from the outer region is discarded.

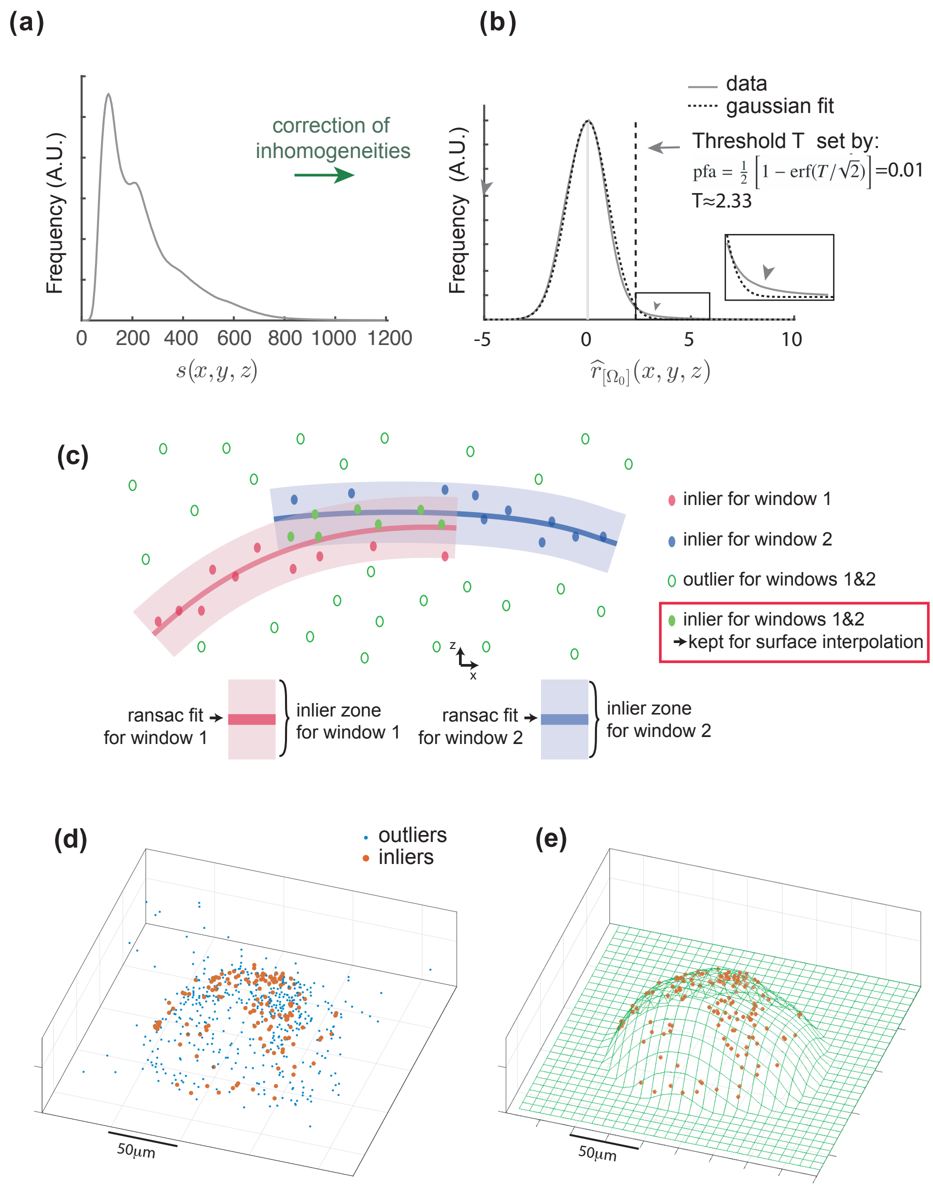

Figure S2. Estimation of a surface of interest of a wing imaginal disc epithelium, from a fractional sampling of 0.1% of the space using the same data as figure 2a-c. (a) Intensity-histogram of the full stack image. (b) After correction of inhomogeneities estimated using only 0.1% of the data, the histogram of full stack image collapses on a heavy-tailed Gaussian centered around 0 (dashed black line). Note that histograms of the full stack image are shown to validate the correction of inhomogeneities, but cannot be used in the algorithm when only 0.1% of the points have been acquired. The inset provides a zoom on the heavy tail (arrowhead) which corresponds to the bright points. These bright points are detected with a pure significance test where the threshold T is set to have a pfa of 1%. (c) Drawing of the filters applied on the detected bright points. Two overlapping ransac polynomial fits (here 1D for simplicity) are drawn in red and blue. Each ransac fit allows to classify bright points as inliers and outliers, thus performing a first filter. In the overlapping region, we then only keep the bright points that are inliers for both windows. (d) Detected bright points among the 0.1% pre-scanned points: Robust second order polynomial fits on overlapping windows (based on RANSAC) allows to distribute bright points in two categories: the inliers -which are close to the surface of interest- in red on the figure, and the outliers in blue on the figure. (e) The surface of interest is computed with a bi-cubic harmonic spline interpolation of the inlier points. It is transformed into a volume of interest (shell) by defining a thickness.

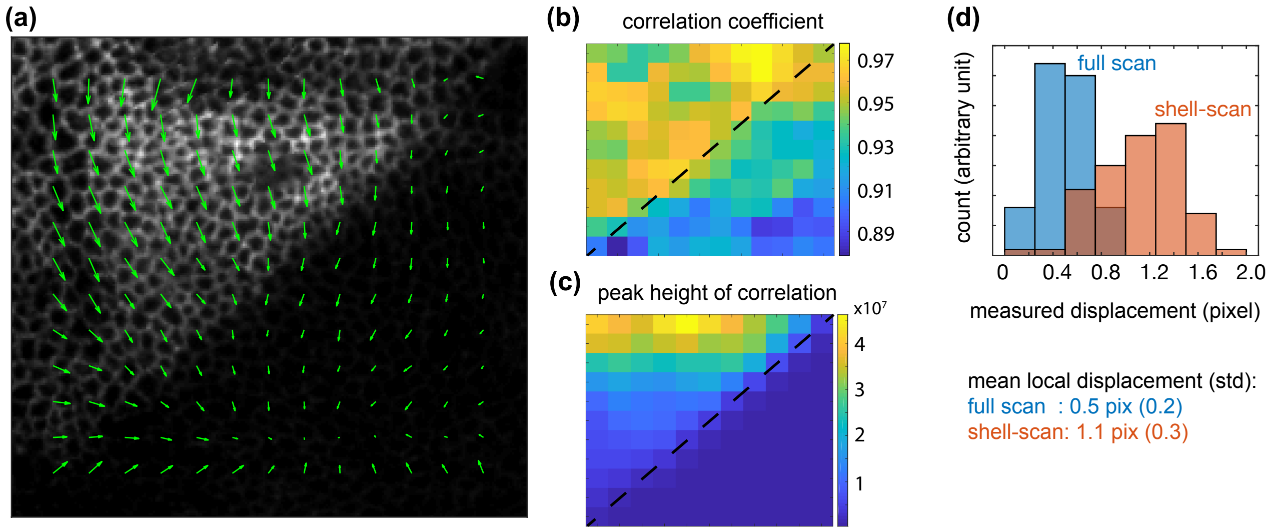

Figure S3. Particle Image velocimetry (PIV) analysis of movements in the hybrid scan acquisition. Two time points spaced by 10 min were used to run a PIV algorithm (PIVlab toolbox[40]). (a) Computed displacement field (green arrows) superposed with the first image used. (b) Correlation coefficient at each interrogation window between the two images used, once the computed displacements have been compensated. (c) Height of the cross correlation peak at each interogation window. (d) Distribution of velocities in the full scan and the shell scan regions.

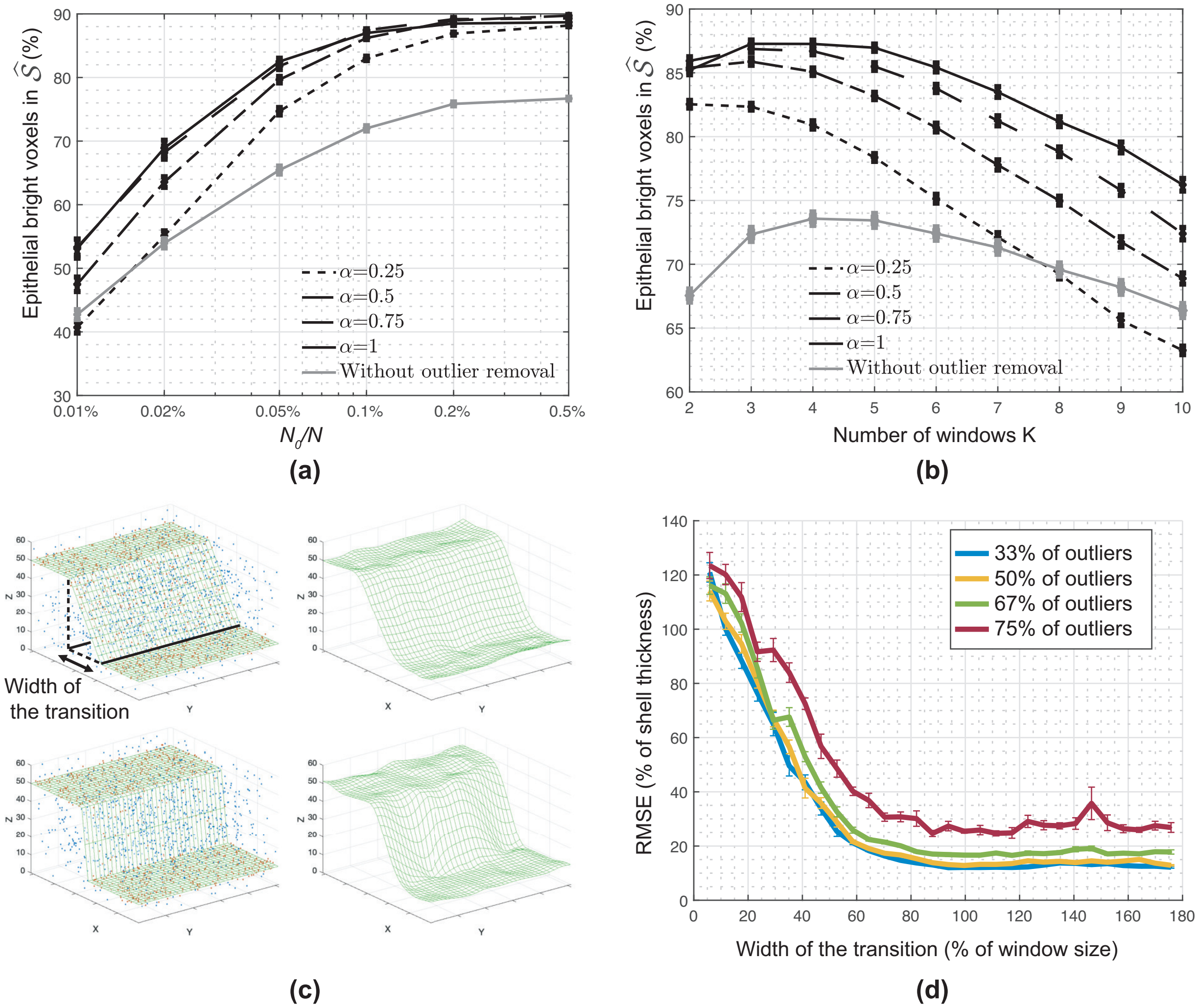

Figure S4. a,b: Percentage of epithelial ground truth bright voxels that are in the estimated thin volume $\widehat{\mathcal{S}}$, obtained on the same sample as in Fig. 4. (a) Evolution as a function of $\eta = N_0/N$ when $\alpha = 1/4$ (dotted line), $1/2$ (dash-dotted line), $3/4$ (dashed line) and 1 (plain black line) and when no outlier removal is used (plain gray line). Results obtained for $K = 3$. (b) Same but as a function of the number K of windows (with size $N_x/K \times N_y/K$) when $\eta = 10^{-3}$. Each curve has been obtained with averaging these percentages for 50 realizations of the set Ω_0 (the error bars correspond to the standard deviation of these percentages). c,d: Robustness of the surface estimation step in the presence of non quadratic surfaces with abrupt transitions. (c) Left: True surfaces generated for 2 values of the transition width between $z = 10$ and $z = 50$ (Top: 88% of the windows' size, Bottom: 18%). The 3D images are composed of $1024 \times 1024 \times 60$ voxels. The surfaces are then estimated from a set of points, composed of 1000 inliers (in red) randomly distributed inside the $3\mu m$-thickness shell around this surface (still assuming a voxel size of $0.27\mu m \times 0.27\mu m \times 0.5\mu m$, see Experimental set-up in the Method section) and of outliers (in blue) randomly and uniformly distributed inside the whole image volume (in this example: 50% of points corresponds to outliers). Right: Corresponding surface estimation results. (d) Evolution of the Root Mean Square Error (along z) between the true surface and the estimated one, as a function of the width of the transition and for different amount of outliers (values averaged on 10 trials). For these experiments, $\alpha = 1$ and $K = 3$ (i.e. windows' width in x,y of 341 pixels).

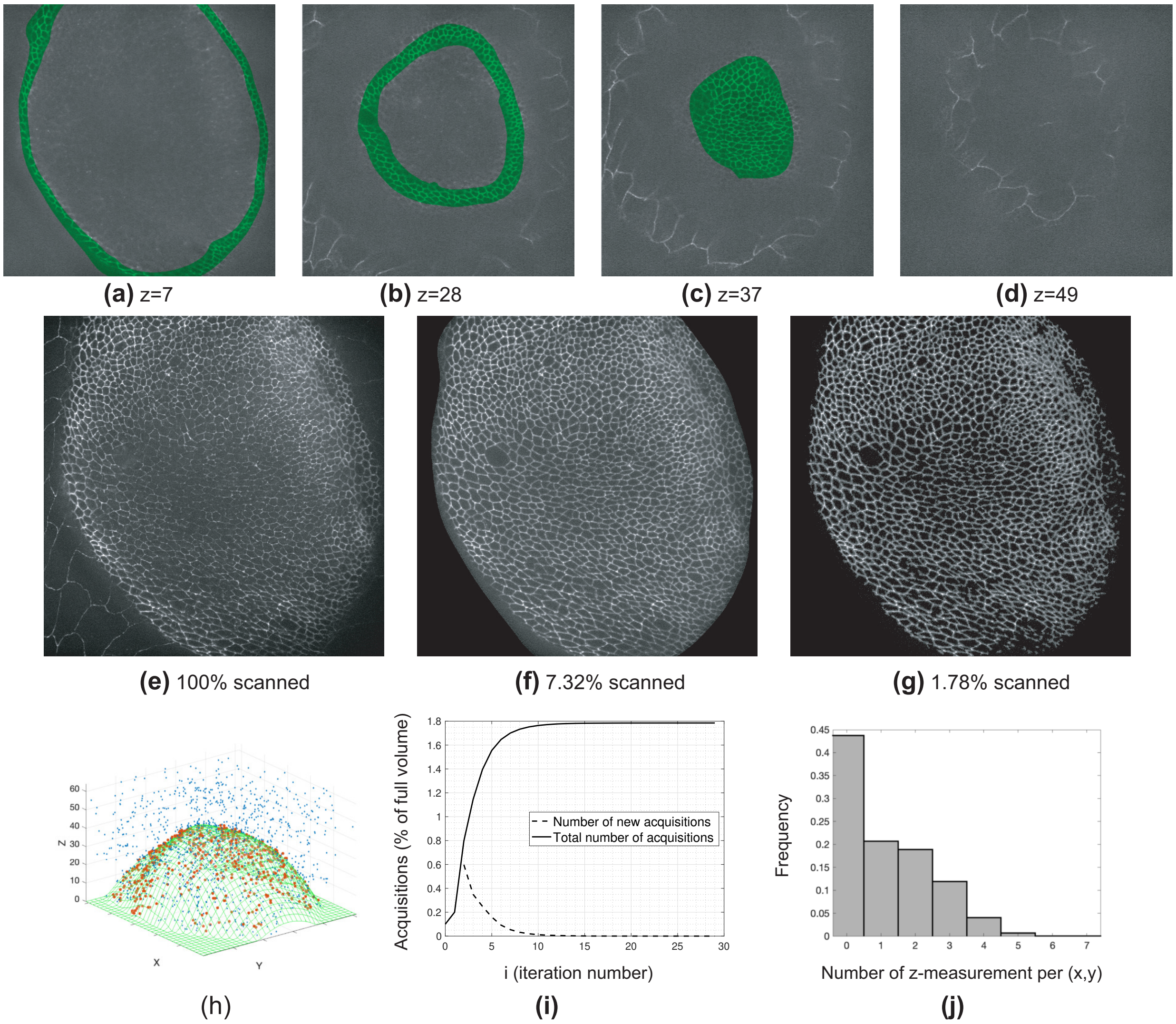

Figure S5. Emulation of shell-scan and propagative-scan on a 3D image of imaginal disc ($1024 \times 1024 \times 64$ voxels) acquired with a spinning disc confocal microscope, using same parameter settings as in the examples shown in the article ($\eta = 0.1\%$, pfa $= 1\%$, $\varepsilon = 3\mu m$, $\alpha = 1$, $K = 3$). (a-d) Overlay of full-scan XY sections of the tissue (gray) with the estimated shell (green) at 4 different z-planes. Imaginal cells are well encapsulated by the shell, while cells of the overlying peripodial membrane are properly discarded. (e-g) Maximum intensity projections along z of intensities $s(x,y,z)$ acquired with full-scan (e), and of normalized intensities $\widehat{r}_{[\Omega_0]}(x,y,z)$ acquired with shell-scan (f) and with propagative-scan with $\beta = 5$ pixels (g). Shell-scan: 7.32% of the volume scanned (11.7% of the tight bounding-box encapsulating only the imaginal disc). Propagative-scan: 1.78% of the volume scanned (2.85% of the tight bounding-box). (h) Surface estimated using the 0.1% pre-scanned points. Red and blue dots correspond to points acquired in the pre-scan that have been detected bright, which have been classified either as inliers (red) or outliers (blue). (i) Evolution of the number of acquisitions with the propagative-scan approach. Convergence is reached in 29 iterations, while almost all acquisitions have been performed after only 15 iterations. (j) Histogram of the number of acquisitions along z performed for each (x,y) location with the propagative-scan approach: For more than 40% of the (x,y), no acquisitions have been performed, and only one acquisition has been done along z on 20% of the (x,y) locations.

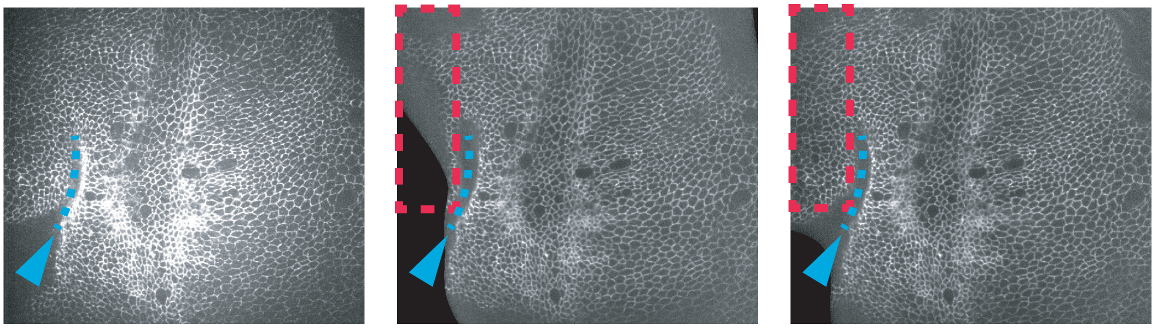

(a) Full-scan
(100% scanned)

(b) Shell-scan (K=3)
(6.1% scanned)

(c) Shell-scan (K=7)
(7% scanned)

Figure S6. Emulation of shell-scan on a more complex 3D image of imaginal disc ($1024 \times 1024 \times 85$ voxels) that presents a surface fold after mechanical tearing (blue arrow and dotted line) and acquired with a spinning disc confocal microscope. (a) Maximum intensity projections along z of intensities $s(x,y,z)$ acquired with full-scan. (b) Corresponding maximum intensity projections along z of normalized intensities $\widehat{r}_{[\Omega_0]}(x,y,z)$ acquired with shell-scan (same parameter settings as in Fig. S5). The tear induced rapid variations of the surface that cannot be easily modeled with second order polynomials on windows of size $1024/K$ when $K = 3$. Consequently, a region of the tissue has not been properly captured (red dotted rectangle in b). A solution can be to reduce the size of the windows, i.e. to increase K. (c) Same as (b) but for $K = 7$. The surface estimated is now more complex, and captures the previously missing part of the tissue (red rectangle in c).

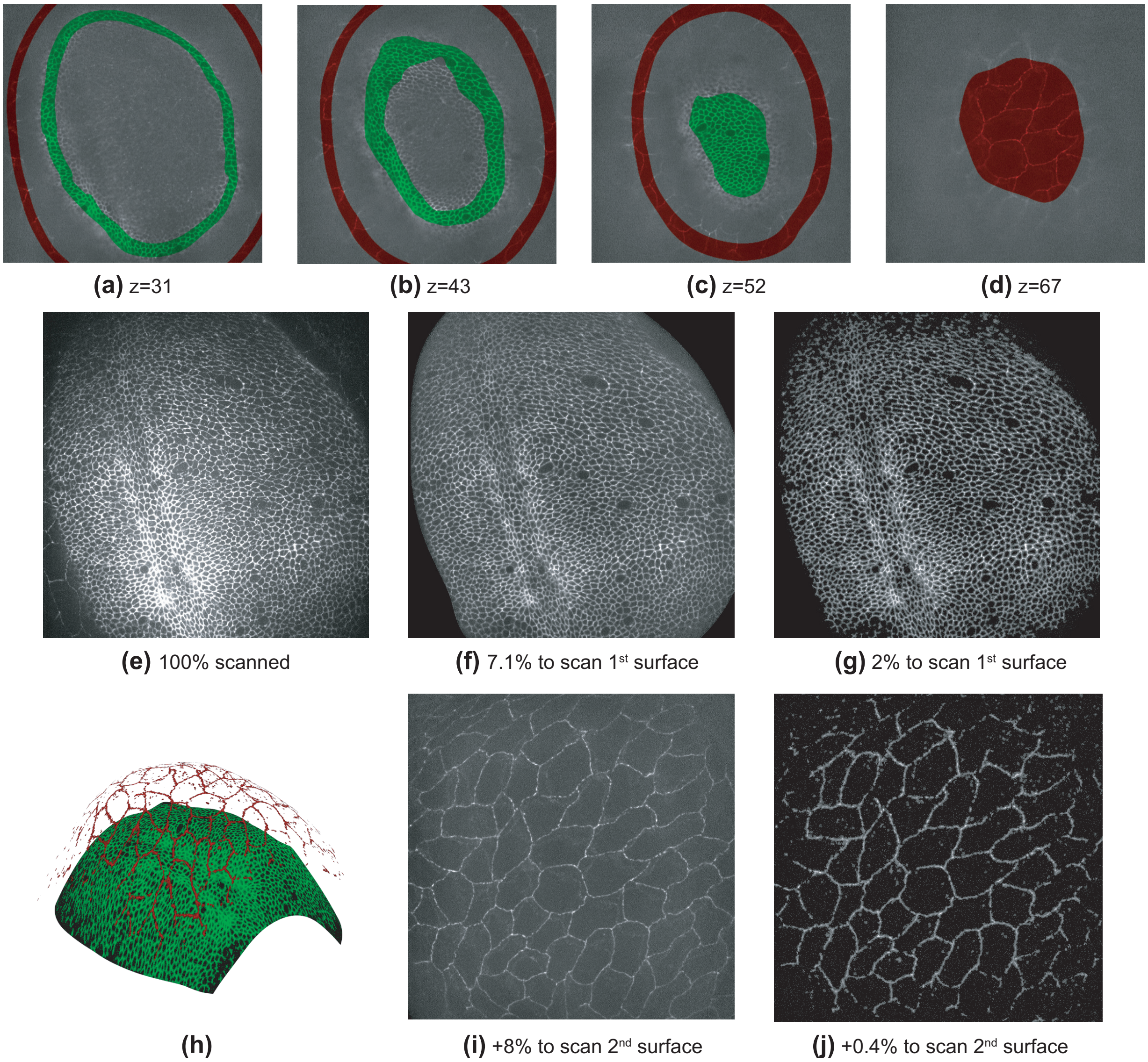

Figure S7. Emulation of a recursive use of shell-scan or propagative-scan to image cell-contours that are located on two different stacked surfaces, obtained on a 3D tissue ($1024 \times 1024 \times 75$ voxels) acquired with a spinning disc confocal microscope. For that purpose, same parameter settings as in the previous example (Fig.S5) are used to estimate the first surface (i.e. the most populated one) and to preform a shell-scan or a propagative-scan inside the corresponding shell. The surface estimation step can then be re-employed to estimate a second surface above the first shell, without requiring other acquisitions than the 0.1% performed during the pre-scan. This second surface being much less populated than the first one (due to larger cell sizes), but with a simpler shape, we set $K = 2$ instead of 3. Shell-scan or propagative-scan can then be used once again to image the corresponding shell. (a-d) Overlay of full-scan XY sections of the tissue (gray) with the two estimated shells (shell 1 in green, shell 2 in red) at 4 different z-planes. (e) Maximum intensity projections along z of intensities $s(x,y,z)$ acquired with full-scan: the cellular contours belonging to the 2 surfaces are mixed. (f,g,i,j) Maximum intensity projections along z of normalized intensities $\widehat{r}_{[\Omega_0]}(x,y,z)$ acquired with shell-scan inside shell 1 (f) and shell 2 (g) and with propagative-scan inside shell 1 (i) and shell 2 (j). (h) 3D-visualisation of the normalized intensities acquired with using twice the propagative-scan. Shell-scan: 7.1% of the volume scanned to image first shell, and 8.0% in addition to image second shell. Propagative-scan: 2.0% of the volume scanned for the first shell, and only 0.4% in addition for second shell.

Supplementary text: Estimation of the surface of interest

Correction of spatial inhomogeneities

As previously mentioned, due mainly to variations of illumination in the sample, the signal $s(x,y,z)$ measured at voxel of coordinates (x,y,z) has to be corrected from inhomogeneities before detecting bright voxels. This correction has to be estimated using only the few voxels acquired in the pre-scan (typically 0.1% of the volume). A simple model of inhomogeneities is thus considered, which relies on a spatially varying multiplicative coefficient $a(x,y,z)$ so that $s(x,y,z) = a(x,y,z)s_0(x,y,z)$ where $s_0(x,y,z)$ is a signal with statistical properties that are constant over the sample space for background voxels (i.e. non-bright voxels).

Thus, assuming $a(x,y,z)$ is a function of x,y,z with slow x and y variations, the spatial averaging of $s(x,y,z)$ on background voxels in a xy-neighborhood should thus be equal to $a(x,y,z)$ (up to a multiplicative constant). To be robust to the presence of non-background bright voxels, instead of using spatial averaging, $a(x,y,z)$ is estimated for each z-plane as the median of $s(x,y,z)$ using only the pre-scanned voxels (x',y',z') with $z' = z$ and (x',y') falling inside a square window of size $W_a \times W_a$ pixels centered on (x,y)[1]. To emphasize the dependency to the set Ω_0, this estimator will be noted $\widehat{a}_{[\Omega_0]}(x,y,z)$.

Since $N_0 \ll N$ (with $N = \text{card}[\Omega]$ and $N_0 = \text{card}[\Omega_0]$), the size W_a of the sliding window is mainly imposed by the typical number of voxels of Ω_0 that will fall inside a $W_a \times W_a$ pixel window. In this paper, we fixed W_a so that each sliding window contains approximately 100 voxels when $N_0/N = 10^{-3}$, leading to $W_a = 317$ pixels.

As $\widehat{a}_{[\Omega_0]}(x,y,z)$ is a slowly varying function of (x,y), one can estimate $\widehat{a}_{[\Omega_0]}$ only every W_a/N_{skip} voxels on x and y and perform a (x,y)-2D cubic spline interpolation in between ($N_{skip} = 5$ throughout this paper). This reduces the number of calls to the computationally intensive median function to shorten computational time.

On background voxels, $s(x,y,z)/\widehat{a}_{[\Omega_0]}$ should now be homogeneous and have a unit mean, and it will also be assumed to be distributed according to a Gaussian probability density function (see histograms in Fig. S2b). The variance σ^2 of this Gaussian is then estimated using only the left side of the histogram, in order to be robust to the presence of bright points:

$$\widehat{\sigma}^2_{[\Omega_0]} = \frac{1}{\text{card}(\Omega_0^-)} \sum_{(x,y,z)\in\Omega_0^-} \left[\frac{s(x,y,z) - \widehat{a}_{[\Omega_0]}(x,y,z)}{\widehat{a}_{[\Omega_0]}(x,y,z)} \right]^2 \tag{2}$$

where $\Omega_0^- = \{(x,y,z) \in \Omega_0 \mid s(x,y,z) \leq \widehat{a}_{[\Omega_0]}(x,y,z)\}$ and card stands for the cardinal - the number of elements of an ensemble. On background voxels, the normalized signal $\widehat{r}_{[\Omega_0]}(x,y,z)$ introduced in equation (1) should thus be distributed according to a Gaussian with zero mean and unit variance, allowing to detect bright points with the following pure-significance test $\widehat{r}_{[\Omega_0]}(x,y,z) > T$ where T is linked to the pfa[35].

Estimation of the epithelial surface

Let us define Ω_0^D the subset of pre-scanned points Ω_0 that are detected to be bright points. They will then be used to estimate the epithelial surface since fluorophores are assumed to be mainly located around the epithelial surface. Nevertheless, the coordinates of these bright points cannot be directly used to interpolate the epithelial surface, since it will be highly corrupted by outliers due to false alarms or more difficult to fluorophores located outside the surface of interest, which can be due for example to the presence of another less populated surface (see Fig. 1a and Fig. 2a-c).

In this paper, it is proposed to use a second order polynomial local averaging of the z-coordinates of the points of Ω_0^D combined with outlier removal before interpolating the epithelial surface. This allows us not only to automatically remove points that are not on the surface of interest but also to denoise the z-coordinates of the interpolating points before interpolation, which is all the more important that Ω_0^D contains few points.

The combined averaging and outlier removal approach relies on local RANSAC[36] (RANdom Sample Consensus) second-order 2D polynomial estimations that will be successively applied on overlapping windows. For an illustration, Fig. S2c provides a drawing to describe 2 overlapping 1D polynomial estimations. More precisely, it will be assumed that the epithelial surface $Z(x,y)$ can be locally approximated with a 2D second order polynomial shape inside a (x,y) neighborhood of size $w_x \times w_y$ pixels, with $w_x = N_x/K$ and $w_y = N_y/K$, where K is an integer and where N_x, N_y, N_z are the sizes in voxels of the full voxel-space Ω along the x, y, z directions (i.e. $\Omega = \{(x,y,z) \in [1,N_x] \times [1,N_y] \times [1,N_y]\}$). In all this paper, we chose $K = 3$, which means that the surface of interest can be approximated with a 2D second order polynomial shape at least on windows with width a third of the image width. We then define $(2K+1)^2$ overlapping windows $W_{i,j}$ with i and j in $[0,1,2,\ldots,2K]$, of size $w_x \times w_y$ and centered on the coordinate $(x,y) = (i\,w_x/2, j\,w_y/2)$. Thus, each $(x,y) \in [1,N_x] \times [1,N_y]$ falls exactly in 4 different windows.

[1] Note that if the measured signal $s(x,y,z)$ presents an offset B (i.e. $s(x,y,z) = a(x,y,z)s_0(x,y,z) + B$), this offset has to be removed before estimating $a(x,y,z)$.

On each of these windows $W_{i,j}$, the coordinates $(x,y,z) \in \Omega_0^D$ so that $(x,y) \in W_{i,j}$ will then be used to estimate a local polynomial fit, based on a robust RANSAC estimator. For that purpose, it is assumed that in $W_{i,j}$, $Z(x,y)$ can be approximated by a second order 2D polynomial

$$F_{\theta_{i,j}}(x,y) = \theta_{i,j}^{(0)} + \theta_{i,j}^{(1)}\, x + \theta_{i,j}^{(2)}\, y + \theta_{i,j}^{(3)}\, xy + \theta_{i,j}^{(4)}\, x^2 + \theta_{i,j}^{(5)}\, y^2 \tag{3}$$

with $\boldsymbol{\theta}_{i,j} = (\theta_{i,j}^{(0)}, \theta_{i,j}^{(1)}, \dots, \theta_{i,j}^{(5)})^T$ the unknown parameter vector of the polynomial fit in window $W_{i,j}$. To estimate $\boldsymbol{\theta}_{i,j}$, RANSAC estimation relies on the assumption that the epithelial surface we are interested in is the most populated surface. It consists in determining the parameter $\widehat{\boldsymbol{\theta}}_{i,j}$ of the surface that perfectly fits 6 points with coordinates (x,y,z) randomly chosen among the sample Ω_0^D with $(x,y) \in W_{i,j}$, and in counting the number of points inside Ω_0^D and $W_{i,j}$ so that $|z - F_{\widehat{\boldsymbol{\theta}}_{i,j}}(x,y)| < \varepsilon/2$. Such points are considered to be inliers, and the other ones outliers. The parameter ε corresponds to the width of the epithelial surface, which may be assumed known by the user and is fixed to $\varepsilon = 3\mu$m throughout this paper. This random process is then iterated n times[2], allowing one to select among these n trials, the estimated surface that provides the greatest set of inliers. Let $\mathcal{I}_{i,j}$ be this set of inliers. The parameter $\widehat{\boldsymbol{\theta}}_{i,j}$ can then be re-estimated in the Least Mean Square (LMS) sense, using all the points inside $\mathcal{I}_{i,j}$.

For each window $W_{i,j}$, using RANSAC estimator then allows one both to estimate a local polynomial fit $F_{\widehat{\boldsymbol{\theta}}_{i,j}}$ and a list of inliers $\mathcal{I}_{i,j}$. Since these windows overlap, each point of Ω_0^D has thus been classified between inlier and outlier on 4 windows (the windows overlapping by half of their size). For an illustration of this, Fig. S2c provides a drawing describing 2 overlapping 1D polynomial estimations.

The 4 classifications being possibly different, we define a tolerance factor α, so that a point $(x,y,z) \in \Omega_0^D$ is considered to be an inlier, if among all the windows containing this point, it has been classified as inlier with a proportion greater than α, i.e. if $n_{\mathcal{I}}(x,y,z)/4 \geq \alpha$ with $n_{\mathcal{I}}(x,y,z)$ the number of time the point of coordinates $(x,y,z) \in \Omega_0^D$ has been classified as inliers (with $n_{\mathcal{I}}(x,y,z) \in \{0,1,2,3,4\}$). This set of inliers will then be denoted $\mathcal{I}$ (see Fig. S2d, obtained with alpha=1). Choosing $\alpha = 1$ means that $\mathcal{I}$ only contains points that have never been classified as outliers. On the contrary, with $\alpha = 1/4$, a point is in $\mathcal{I}$ as soon as it as been classified as inlier at least in one window. The influence of this factor α on the performance of this approach will be studied below.

Thus, for each point $(x,y,z) \in \mathcal{I}$, $n_{\mathcal{I}}(x,y,z)$ polynomial denoised surfaces $F_{\widehat{\boldsymbol{\theta}}_{i,j}}(x,y)$ have been estimated (with (i,j) so that $(x,y,z) \in \mathcal{I}_{i,j}$). A refined z estimate $\widehat{z}(x,y)$ at this location (x,y) is then defined as the averaging of these $n_{\mathcal{I}}(x,y,z)$ estimates $F_{\widehat{\boldsymbol{\theta}}_{i,j}}(x,y)$, allowing in particular to smooth surfaces at window interfaces.

To summarize the steps of surface estimation so far, we have removed outliers and have denoised the z-coordinate of the remaining bright points by replacing their (x,y,z) coordinates with $(x,y,\widehat{z}(x,y))$, where $\widehat{z}(x,y)$ has been estimated using robust polynomial fits and averaging among overlapping windows. A surface $\widehat{Z}(x,y)$ can then be estimated at every point of the image using a simple 3D interpolation from the points $(x,y,\widehat{z}(x,y))$, $(x,y) \in \mathcal{I}$ (see Fig. S2e). We use bi-cubic harmonic spline interpolation[37], but other choices could be envisaged.

This 3D fit $\widehat{z}$ allows one to define the set of voxels where the bright structure of interest lies, defined as

$$\widehat{\mathcal{S}} = \left\{ (x,y,z) \text{ so that } \left| z - \widehat{Z}(x,y) \right| \leq \varepsilon/2 \right\} \tag{4}$$

where ε still represents the width of the thin epithelial volume.

To complete the quantitative analysis shown on Fig. 4c, we study in Fig. S4a,b the influence of the parameters α and K on the performance of this epithelial surface estimation approach. The full stack 3D image of this biological sample has been acquired using the experimental setup of Fig. 1c, in order to determine the ground truth epithelial shell $\mathcal{S}_0$ (defined like $\widehat{\mathcal{S}}$ but using the ground truth surface instead of the estimated one), and to emulate this surface estimation strategy with different parameter settings. As in Fig. 4c, the quality of the surface estimation is quantified with the percentage of bright voxels in $\mathcal{S}_0$ that are effectively present in the estimated epithelial volume $\widehat{\mathcal{S}}$. Each point on the graphs is the averaging of the percentages obtained with 50 different pre-scans, each leading to a slightly different thin volume $\widehat{\mathcal{S}}$.

Fig. S4.a shows the evolution of this percentage as a function of $\eta = N_0/N$ for different values of the tolerance factor α and when no outlier removal is used (grey curve), and Fig. S4b as a function of the number K of windows used. This shows that the use of a technique robust to the presence of outliers is a key point. Moreover, in Fig. S4a, using a tolerance factor $\alpha = 1$ or $\alpha = 3/4$ leads to the best results (with almost same performance). It can also be noticed that, of course increasing η improves

[2] Practically, n is set so that the probability that at least one trial among the n trials does not contain outlier, is greater than p[36]. In this paper, it has been chosen $p = 0.999$.

the quality of the results, but $\eta = 0.1\%$ leads to a good trade-off between quality of the results and number N_0 of acquisitions performed. Furthermore, it can also be seen in Fig. S4b that $\alpha = 1$ highly increases the robustness to the choice of K. In all this paper, one will thus use $\alpha = 1$ and windows with width a third of the image width (i.e. $K = 3$).

As the surface estimation step relies on local second order polynomial estimations, its robustness to the presence of non quadratic surfaces with abrupt transitions is investigated in Fig. S4 (second row). For that purpose, we generate a surface composed of two horizontal surfaces, located at height $z = 50$ on the left part of the image and $z = 10$ on the right part (for a total x, y, z volume of $1024 \times 1024 \times 60$ voxels) and connected by a linear surface (see Fig. S4c). The surface is then estimated from a set of points, composed of 1000 inliers randomly distributed inside the $3\mu m$-thickness shell around this surface (still assuming a voxel size of $0.27\mu m \times 0.27\mu m \times 0.5\mu m$, see Experimental set-up in the Method section). Moreover, to assess also for the robustness of the proposed approach to a high number of outliers, outliers randomly and uniformly distributed inside the whole image volume are added to this initial set of inliers: simulations have then been performed when 1/3, 1/2, 2/3 and 3/4 of the points in the volume corresponds to outliers. The evolution of the Root Mean Square Error (along z) between this surface and the estimated one is then plotted on Fig. S4d as a function of the width of the transition between the two horizontal planes: the more abrupt the transition, the less it can be modeled by second order polynomials. Since the capabilities to model this transition mainly depends on the size of the windows used to locally estimate quadratic fits, the size of the transition along x is expressed as a percentage of the window size (which corresponds to a third of the image in Fig. S4c and d, i.e. 341 pixels, since $K = 3$).

On this example, the surface estimation step is relatively robust to such abrupt changes, provided the transition is smaller that 60% of the window size. Moreover, increasing the number of outliers does not deteriorate the performance of the surface estimation step, even when 2/3 of the points correspond to outliers. Performance begins to degrade only when 75% of the points corresponds to outliers, which confirms the interest of this approach in presence of a high number of outliers.

References

1. Cutrale, F., Fraser, S. E. & Trinh, L. A. Imaging, visualization, and computation in developmental biology. *Annual Review of Biomedical Data Science* **2**, 223–251 (2019).
2. Gao, R. X. *et al.* Cortical column and whole-brain imaging with molecular contrast and nanoscale resolution. *Science* **363**, eaau8302 (2019).
3. Mertz, J. Introduction to optical microscopy. 2nd edn. (Cambridge: Cambridge University Press, 2019).
4. Mertz, J. Optical sectioning microscopy with planar or structured illumination. *Nature Methods* **8**, 811–819 (2011).
5. Carlton, P. M. *et al.* Fast live simultaneous multiwavelength four-dimensional optical microscopy. *Proceedings of the National Academy of Sciences of the United States of America* **107**, 16016–16022 (2010).
6. Hoebe, R. A. *et al.* Controlled light-exposure microscopy reduces photobleaching and phototoxicity in fluorescence live-cell imaging. *Nature Biotechnology* **25**, 249–253 (2007).
7. Chu, K. K., Lim, D. & Mertz, J. Enhanced weak-signal sensitivity in two-photon microscopy by adaptive illumination. *Optics Letters* **32**, 2846–2848 (2007).
8. Staudt, T. *et al.* Far-field optical nanoscopy with reduced number of state transition cycles. *Optics Express* **19**, 5644–5657 (2011).
9. Dreier, J. *et al.* Smart scanning for low-illumination and fast RESOLFT nanoscopy in vivo. *Nature Communications* **10**, 1–11 (2019).
10. Vinçon, B., Geisler, C. & Egner, A. Pixel hopping enables fast STED nanoscopy at low light dose. *Optics Express* **28**, 4516–4528 (2020).
11. Caarls, W. *et al.* Minimizing light exposure with the programmable array microscope. *Journal of Microscopy* **241**, 101–110 (2011).
12. Chakrova, N. *et al.* Adaptive illumination reduces photobleaching in structured illumination microscopy. *Biomedical Optics Express* **7**, 4263–4274 (2016).
13. Guillot, C. & Lecuit, T. Mechanics of epithelial tissue homeostasis and morphogenesis. *Science* **340**, 1185–1189 (2013).
14. LeGoff, L., Rouault, H. & Lecuit, T. A global pattern of mechanical stress polarizes cell divisions and cell shape in the growing *Drosophila* wing disc. *Development* **140**, 4051–4059 (2013).
15. Heemskerk, I. & Streichan, S. J. Tissue cartography: compressing bio-image data by dimensional reduction. *Nature Methods* **12**, 1139–1142 (2015).
16. Heemskerk, I., Lecuit, T. & LeGoff, L. Dynamic clonal analysis based on chronic in vivo imaging allows multiscale quantification of growth in the *Drosophila* wing disc. *Development* **141**, 2339–2348 (2014).
17. Heller, D. *et al.* Epitools: an open-source image analysis toolkit for quantifying epithelial growth dynamics. *Developmental Cell* **36**, 103–116 (2016).
18. de Reuille, P. B. *et al.* Morphographx: A platform for quantifying morphogenesis in 4D. *eLife* **4**, e05864 (2015).
19. Goldenberg, G. & Harris, T. J. C. Adherens junction distribution mechanisms during cell-cell contact elongation in *Drosophila*. *PLoS One* **8**, e79613 (2013).
20. Supatto, W. *et al.* In vivo modulation of morphogenetic movements in *Drosophila* embryos with femtosecond laser pulses. *Proceedings of the National Academy of Sciences of the United States of America* **102**, 1047–1052 (2005).
21. Koester, H. J. *et al.* Ca^{2+} fluorescence imaging with pico-and femtosecond two-photon excitation: signal and photodamage. *Biophysical Journal* **77**, 2226–2236 (1999).
22. Hopt, A. & Neher, E. Highly nonlinear photodamage in two-photon fluorescence microscopy. *Biophysical Journal* **80**, 2029–2036 (2001).
23. Schmidt, E. & Oheim, M. Infrared excitation induces heating and calcium microdomain hyperactivity in cortical astrocytes. *Biophysical Journal* **119**, 2153–2165 (2020).
24. Godaliyadda, G. M. *et al.* A framework for dynamic image sampling based on supervised learning . *IEEE Transactions on Computational Imaging* **4**, 1–16 (2018).
25. Hujsak, K. A. *et al.* High speed/low dose analytical electron microscopy with dynamic sampling. *Micron* **108**, 31–40 (2018).

26. Scherf, N. & Huisken, J. The smart and gentle microscope. *Nature Biotechnology* **33**, 815–818 (2015).

27. Strobl, F., Schmitz, A. & Stelzer, E. H. K. Improving your four-dimensional image: traveling through a decade of light-sheet-based fluorescence microscopy research. *Nature Protocols* **12**, 1103–1109 (2017).

28. Débarre, D. *et al.* Mitigating phototoxicity during multiphoton microscopy of live *Drosophila* embryos in the 1.0–1.2 μm wavelength range. *PLoS One* **9**, e104250 (2014).

29. Weigert, M. *et al.* Content-aware image restoration: pushing the limits of fluorescence microscopy. *Nature Methods* **15**, 1090–1097 (2018).

30. Xiao, S. *et al.* Video-rate volumetric neuronal imaging using 3d targeted illumination. *Scientific Reports* **8**, 7921 (2018).

31. Müller, C. B. & Enderlein, J. Image scanning microscopy. *Physical Review Letters* **104**, 198101 (2010).

32. York, A. G. *et al.* Resolution doubling in live, multicellular organisms via multifocal structured illumination microscopy. *Nature Methods* **9**, 749–754 (2012).

33. Wu, J. J. *et al.* Resolution improvement of multifocal structured illumination microscopy with sparse bayesian learning algorithm. *Optics Express* **26**, 31430–31438 (2018).

34. Chakrova, N., Rieger, B. & Stallinga, S. Development of a DMD-based fluorescence microscope. Proceedings of SPIE 9330, Three-Dimensional and Multidimensional Microscopy: Image Acquisition and Processing XXII. San Francisco: SPIE, 2015, 933008.

35. Garthwaite, P. H., Jolliffe, I. T., Jolliffe, I. & Jones, B. *Statistical inference. 2nd edn.* (Oxford: University Press on Demand, 2002).

36. Fischler, M. A. & Bolles, R. C. Random sample consensus: a paradigm for model fitting with applications to image analysis and automated cartography. *Communications of the ACM* **24**, 381–395 (1981).

37. Sandwell, D. T. Biharmonic spline interpolation of geos-3 and seasat altimeter data. *Geophysical Research Letters* **14**, 139–142 (1987).

38. Huang, J. *et al.* Directed, efficient, and versatile modifications of the *Drosophila* genome by genomic engineering. *Proceedings of the National Academy of Sciences of the United States of America* **106**, 8284–8289 (2009).

39. Beira, J. V. & Paro, R. The legacy of *Drosophila* imaginal discs. *Chromosoma* **125**, 573–592 (2016).

40. Thielicke, W. & Sonntag, R. Particle Image Velocimetry for MATLAB: Accuracy and enhanced algorithms in PIVlab. *Journal of Open Research Software* **9**, 10.5334/jors.334 (2021).
